# Attractive targeted sugar bait: the pyrrole insecticide chlorfenapyr and the anti-malarial pharmaceutical artemether–lumefantrine arrest *Plasmodium falciparum* development inside wild pyrethroid-resistant *Anopheles gambiae* s.s. mosquitoes

**DOI:** 10.1186/s12936-023-04758-1

**Published:** 2023-11-09

**Authors:** Raphael N’Guessan, Soromane Camara, Mark Rowland, Ludovic P. Ahoua Alou, Rosine Z. Wolie, Marius G. Zoh, Brou N’Guessan, Innocent Z. Tia, Welbeck A. Oumbouke, Matthew B. Thomas, Alphonsine A. Koffi

**Affiliations:** 1 Institut Pierre Richet (IPR), Institut National de Santé Publique (INSP), Bouaké, Côte d’Ivoire; 2 Vector Control Product Evaluation Centre (VCPEC)-Institut Pierre Richet (VCPEC-IPR)/INSP, Bouaké, Côte d’Ivoire; 3 Department of Disease Control, London School of Hygiene and Tropical Medicine, London, UK; 4 Université Nangui Abrogoua, UFR Des Sciences de la Nature, Abidjan, Côte d’Ivoire; 5 Innovative Vector Control Consortium, IVCC, Liverpool, UK; 6 Department of Entomology & Nematology, The University of Florida, Gainesville, FL USA

**Keywords:** *Anopheles gambiae* s.s., Chlorfenapyr, Attractive targeted sugar baits, Oocyst prevalence, Oocyst intensity, Vector control

## Abstract

**Background:**

Attractive targeted sugar bait (ATSB) is a novel approach to vector control, offering an alternative mode of insecticide delivery via the insect alimentary canal, with potential to deliver a variety of compounds new to medical entomology and malaria control. Its potential to control mosquitoes was recently demonstrated in major field trials in Africa. The pyrrole chlorfenapyr is an insecticide new to malaria vector control, and through its unique mode of action—disruption of ATP mediated energy transfer in mitochondria—it may have direct action on energy transfer in the flight muscle cells of mosquitoes. It may also have potential to disrupt mitochondrial function in malarial parasites co-existing within the infected mosquito. However, little is known about the impact of such compounds on vector competence in mosquitoes responsible for malaria transmission.

**Methods:**

In this study, ATSBs containing chlorfenapyr insecticide and, as a positive control, the anti-malarial drugs artemether/lumefantrine (A/L) were compared for their effect on *Plasmodium falciparum* development in wild pyrethroid-resistant *Anopheles gambiae* sensu stricto (s.s.) and for their capacity to reduce vector competence. Female mosquitoes were exposed to ATSB containing either sublethal dose of chlorfenapyr (CFP: 0.025%) or concentrations of A/L ranging from 0.4/2.4 mg/ml to 2.4/14.4 mg/ml, either shortly before or after taking infective blood meals. The impact of their component compounds on the prevalence and intensity of *P. falciparum* infection were compared between treatments.

**Results:**

Both the prevalence and intensity of infection were significantly reduced in mosquitoes exposed to either A/L or chlorfenapyr, compared to unexposed negative control mosquitoes. The A/L dose (2.4/14.4 mg/ml) totally erased *P. falciparum* parasites: 0% prevalence of infection in female mosquitoes exposed compared to 62% of infection in negative controls (df = 1, χ^2^ = 31.23 p < 0.001). The dose of chlorfenapyr (0.025%) that killed < 20% females in ATSB showed a reduction in oocyte density of 95% per midgut (0.18/3.43 per midgut).

**Conclusion:**

These results are evidence that chlorfenapyr, in addition to its direct killing effect on the vector, has the capacity to block *Plasmodium* transmission by interfering with oocyte development inside pyrethroid-resistant mosquitoes, and through this dual action may potentiate its impact under field conditions.

## Background

Current vector control relies primarily on the use of long-lasting insecticidal nets (LLINs) and indoor residual spraying (IRS). In sub-Saharan Africa, the up-scaling of these control interventions has resulted a decline in malaria burden over the last two decades [1]. Despite the progress achieved [2, 3], elimination remains distant prospect, made worse by the spread of insecticide resistance across *Anopheles* mosquito species, which is undermining the global effort [4–6]. To maintain momentum and overcome insecticide resistance, the shortfalls of the current interventions need to be addressed with new, complementary vector control tools, one of which is targeted sugar bait [7].

Attractive targeted sugar bait (ATSB) is a novel vector control tool that exploits the need for both male and female mosquitoes to take sugar meal while drawing on insecticide more typically used in agriculture [8]. There have been several successful attempts to control mosquito populations using ATSB outdoors [9–12]. Indoor use of ATSB is effective too, as indicated in experimental hut trials that demonstrated indoor use of ATSB in combination with an untreated bed net or LLINs can induce high levels of *Anopheles gambiae* mortality in Côte d’Ivoire and Tanzania [13, 14]. A large-scale field trial of ATSB conducted in Mali, has significantly reduced malaria parasite transmission by reducing the number of older females and the number of sporozoite infected females [15]. So far, almost all studies on toxic sugar bait are evaluations of their gross effects, the aim being to reduce the longevity the wild mosquitoes. The potential effect of toxic sugar bait on the parasite development inside wild mosquitoes has received scant attention. Whether toxic sugar bait could affect parasite development inside wild resistant mosquitoes is worthy of further study in this era of high insecticide resistance. Indeed mosquito populations that are the most dangerous for humans and of greatest epidemiological relevance are insecticide-resistant infective mosquitoes [16].

Numerous studies have investigated potential effects of insecticides and insecticide resistance on vector competence or effect of *Plasmodium* infection on mosquito behaviour. A recent study demonstrated a cost of *Plasmodium* infection on mosquito survival at transmissible stages of infection [17]. According to Thievent et al. [18], infection reduces personal protection offered by insecticide-treated nets (ITNs). Another study found that infection can partially restore susceptibility to insecticide among mosquitoes carrying resistance alleles [19]. In some studies, exposure to deltamethrin, dichloro-diphenyl-trichloroethane (DDT) or bendiocarb insecticides inhibited development of *Plasmodium falciparum* in insecticide-resistant *An. gambiae* sensu stricto (s.s.) [20, 21]. However, other studies found no effect of insecticides on parasite development in mosquitoes [22].

A recent study published in the journal Nature tested the hypothesis that the use of anti-malarial compounds might clear *Plasmodium* infections directly in the *Anopheles* [23]. The authors demonstrated that the development of *P. falciparum* can be completely blocked when female *An. gambiae* mosquitoes take up low concentration of the anti-malarial atovaquone [23]. Almost all of these studies have used insecticide delivery methods that involve mosquito contact with insecticides on bed net or test paper [20, 21, 23].

ATSB constitutes a novel means of deploying insecticide against mosquitoes and has the advantage of being able to utilize a wide range of compounds developed for crop pests. Besides tarsal contact, the toxins are administrable by ingestion and absorbed through the crop or midgut [13, 14]. A major three-centre trial is currently underway in Mali, Kenya, and Zambia [24].

Oocyst formation in the mosquito midgut is a critical stage in the development of the malaria parasite and thus further spread of the pathogen through saliva [25]. Because fewer *P. falciparum* ookinetes successfully cross the midgut epithelium to form oocysts [25, 26], it is therefore possible that ATSB ingestion by the mosquito could cause full parasite arrest in the midgut, and prevent transmission via infective salivary glands.

In this study, it was investigated whether ATSBs containing insecticide or anti-malarial drugs could be an appropriate means to block *P. falciparum* development in wild pyrethroid-resistant *An. gambiae* s.s. from Côte d’Ivoire.

## Methods

### Mosquito collection and rearing

*Anopheles gambiae* sensu lato (s.l.) larvae were collected from breeding sites in rice fields around the experimental station of Mbe. Adult and larval mosquitoes were maintained in a purpose-built insectary at 27 ± 2 °C and 80% relative humidity with a light: dark photoperiod of 12:12 h. Larvae were reared in groups of about 400 in 1.5 l of distilled water and were fed TetraMin® fish food. Adults were fed a 10% sugar solution and took up blood meal on volunteers’ feet. Mosquitoes used for this study were *An. gambiae* s.s. G2 strain, resistant to pyrethroids, DDT, and carbamates with mechanisms that include mixed function oxidases (MFOs), esterases and voltage-gated sodium channel (VGSC) point mutations [27, 28].

### *Plasmodium falciparum* experimental infection

Experimental infections were performed as described by Bousema et al. [29]. Ethical approval was obtained from the Ministry of Health in Côte d’Ivoire through the National Ethic Committee of life Sciences and Health N° 023-22/MSHPCMU/CNESVS-km. All human volunteers were enrolled after receipt of written informed consent from their legal guardians. Screening for *P*. *falciparum* infectious human carriers were conducted in primary school groups in Bouaké, Côte d’Ivoire. Eligible children were driven to the laboratory after health examination and gametocyte-containing blood was collected. The blood was centrifuged and then the plasma was removed and replaced with European naïve AB serum. This procedure avoids natural transmission-blocking immune factors [30]. The 3–5 days old mosquitoes were allowed to feed through pre-warmed (37 °C) membrane feeders. After one hour of exposure, unfed females were discarded and only fully fed mosquitoes were kept and maintained in the same conditions as during the rearing. Fed females were given the opportunity to oviposit. This procedure was repeated nine times, each feeding assay using a different gametocyte-infected blood source. All participants carrying *Plasmodium* species were treated with anti-malarial drugs according to the national guidelines.

### ATSB laboratory bioassays

#### Compound exposures and preparation of ATSB solution

The Attractive Sugar Bait (ASB) solution was based on a recipe of 35% guava juice purchased locally in supermarket, 10% sugar solution, 2% orange food dye also bought locally in same supermarket; guava juice is known to be a strong attractant for *An. gambiae* s.l. [11]. The ATSB solution contained chlorfenapyr (Phantom SC 21.45%, BASF) concentrations of 0.0025–0.5% as the toxin [13, 14]. Chlorfenapyr is a pro-insecticide, activated by cytochrome P450s within the insect, that was shown to be effective in ATSB in previous studies [14]. This insecticide shows no cross resistance to common insecticide classes, is effective against pyrethroid-resistant mosquitoes [31, 32].

The ATSB solution also contained anti-malarial compound artemether/lumefantrine 80/480 mg tablets (Plasmocid, CIPHARM), indicated for the treatment of uncomplicated cases of malaria due to *P. falciparum* in adults. Concentrations of A/L ranging from 0.4 /2.4 mg/ml to 2.4/14.4 mg/ml were incorporated into ASB. Artemisinin derivatives are endoperoxides that bind to haem in the digestive vacuole of the parasite. This interaction is believed to cause the release of free radicals that are toxic to the cellular constituents [33]. Preliminary bioassays performed with these anti-malarial drugs showed no impact on mosquito survival during the 2 weeks of testing.

#### ATSB bioassay

Bioassays were performed as described by Furnival‑Adams et al. [13]. ATSB solution (25 ml) was soaked into cotton wool pads and inserted into testing cages. Fifty female mosquitoes, 4–5 days old were denied access to sugar for six hours and then introduced into cages (30 cm × 30 cm × 30 cm). Mosquitoes were left overnight to interact with the ATSB solution. The following morning, mosquitoes were blood fed on gametocyte-infected blood by Direct Membrane Feeding Assay (DMFA) methods.

In the second test, mosquitoes were exposed to ATSB solution 24 h after feeding an infected blood meal. For both tests, 25 ml of ASB (without insecticide or anti-malarial drug) was used as a negative control. Female mosquitoes exposed to ATSB before or after infection were held under standard insectary conditions on a 10% sucrose solution.

#### Oocyst counting

Mosquitoes were maintained for 6 to 7 days in the insectary. Then, midguts were dissected in 0.4% mercurochrome (Sigma-Aldrich), and the infection prevalence and intensity of each individual female was determined by presence and number of oocysts, under a light microscope.

### Statistical analysis

Infection data consisted of two response variables: The prevalence of infection that were calculated by dividing the number of infected mosquitoes (those with one or more oocysts) by the number of dissected mosquitoes and the intensity of infection expressed by the number of oocysts per infected mosquito midgut.

Statistical analysis for the comparison of prevalence of infection and the mean oocyst intensity between treatments were performed using R software version 4.1.2, and figures with GraphPad Prism 7 software. Malaria drug compound and chlorfenapyr datasets were analysed using the same statistical method. The prevalence of infection were compared using a binomial mixed effect model (function “glmer” from the package lme4). The fixed variables were the treatment. The replicate were considered as a random intercept to adjust for sampling variations. For oocyst intensity, differences in the number of oocysts in mosquito midgut of both intervention and negative controls groups were analysed using a Mann–Whitney rank sum test.

## Results

*Anopheles gambiae* s.s. mosquitoes, 3–5 days old, resistant to insecticides were experimentally infected with *P. falciparum*-containing blood collected in human participants in Bouaké, Côte d’Ivoire. Prevalence of infection varied between 61 and 72% across infectious blood samples from distinct blood donors. Overall, very low oocyst loads among blood fed female mosquitoes were observed. Dissection at 6–7 days post-infective blood meal showed oocyst numbers per female ranging from 1 to 50 across experimental infections.

### Effect of anti-malarial drug on *Plasmodium falciparum* development

**Fig. 1:**
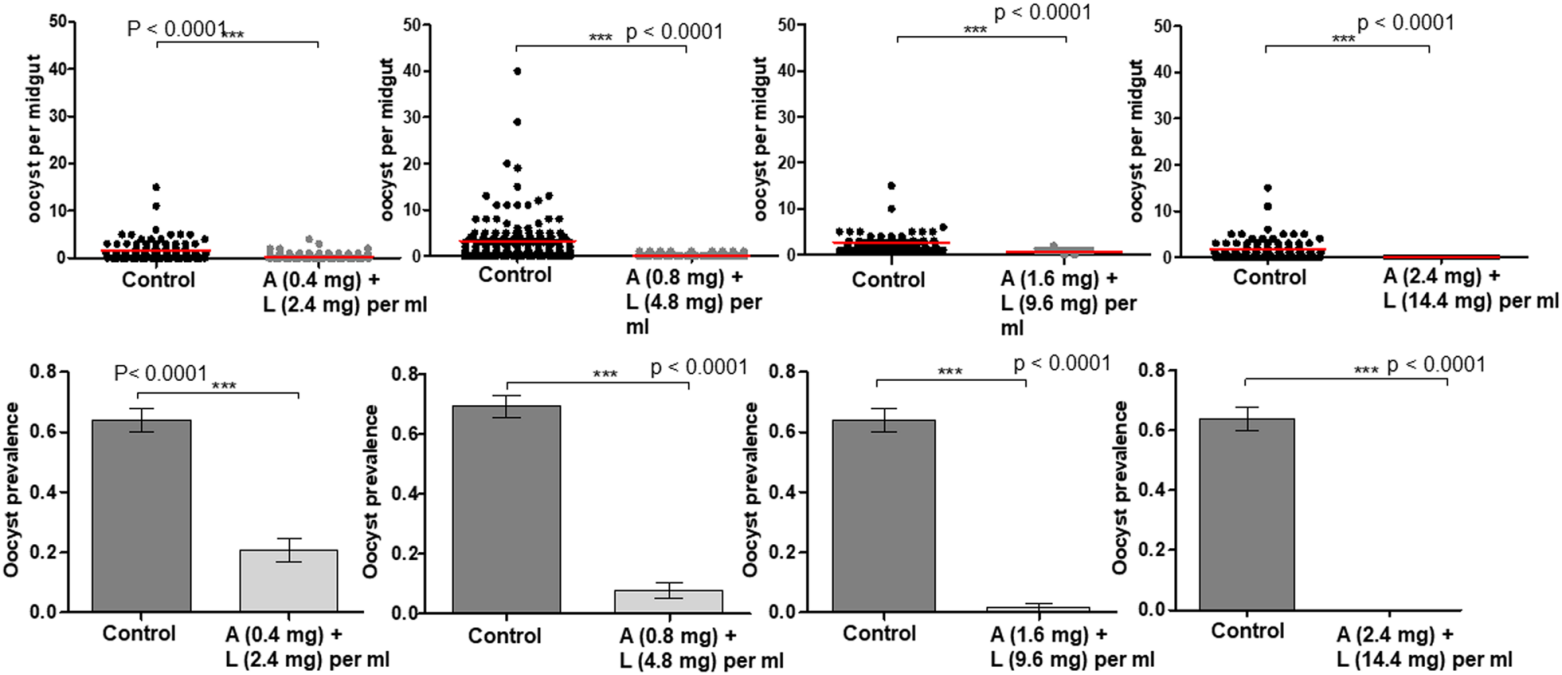
Oocyst burden and prevalence of infected-females and exposed to differents doses of Artemether + Lumefantrine. A + L = Artemether + Lumefantrine, number of oocysts per female midgut is presented as a scatter dot plot for each treatment, Oocyst prevalence is presented as the bar chart, Results are presented as mean ± standard error (s.e)

The first experiment investigated whether *Plasmodium falciparum* development was affected in female mosquitoes exposed to a range of anti-malarial drug concentrations 24 h after initial infection. The results showed a significant dose-dependent protective effect against infection. The prevalence and intensity of infection were lower in mosquitoes that were exposed to anti-malarial ATSB compared with the non-exposed negative control, irrespective of concentration tested (Fig. 1): the lowest dose of A/L (0.4/2.4 mg/ml) induced 61.3% reduction in the prevalence of oocysts (24% [16.1–34.9] for ATSB exposure compared with 62% [51.1–71.7] prevalence in the control, OR [95% CI] = 0.19 [0.1–0.3], p < 0.0001) (Fig. 1); it also decreased significantly the number of oocyst in mosquitoes exposed to A/L (mean of 0.32 ± 0.07 oocysts/midgut) compared with 1.65 ± 0.26 in the control (df = 1, U = 2202, p < 0.0001 ) (Fig. 1). The highest dose (2.4/14.4 mg/ml) presented completely eliminated *P. falciparum* parasites: 0% prevalence infection in female mosquitoes exposed compared with 62% [51.1–71.7] prevalence of oocyst infection in the negative control (Fig. 1).

**Fig. 2:**
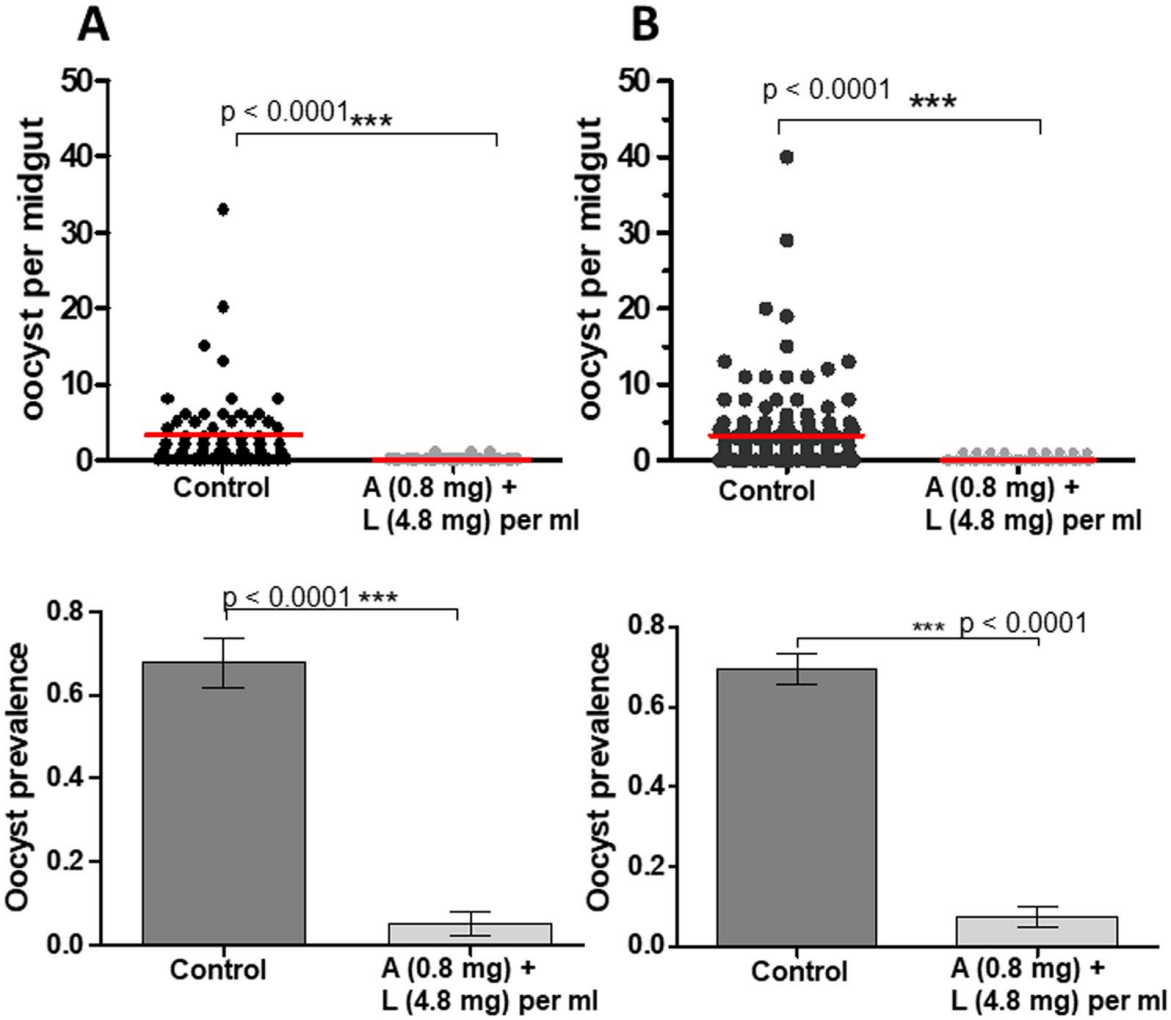
Prevalence of infection and oocyst intensity in *An. gambiae* s.s. exposed to ATSB solution (Artemether + Lumefantrine incorporated into ATSB) 15 h pre-infection (**A**) and 24 h post infection (**B**). A + L = Arthemether + Lumefantrine. Number of oocysts per female midgut is presented as a scatter dot plot for each treatment. Oocyst prevalence is presented as the bar chart, Results are presented as mean ± standard error (s.e)

The next experiment investigated the timing of delivery of ATSB to the infected mosquitoes. The dosage was fixed at a low concentration of A/L (0.8/4.8 mg/ml) and batches of mosquitoes were then exposed mosquitoes to a selected low dose of A/L (0.8/4.8 mg/ml), either 15 h before or 24 h after exposing the mosquitoes to infectious blood meal and analysing the effect on oocyst prevalence and oocyst load. In mosquitoes exposed to this dose of A/L, oocyst prevalence (5% [1.6–14.5]) was significantly lower than that in control (68% [55.1–78.3], OR [95% CI] = 0.02 [0.01–0.09], p < 0.0001) (Fig. 2). The oocyst intensity was also significantly reduced in exposed female (mean of 0.05 ± 0.03 oocysts/midgut) compared to control (3.43 ± 0.68, U = 642, p < 0.0001) (Fig. 2). The timing of exposure to A/L either 15 h pre-infection or 24 h post-infection made no significant difference to the effectiveness of the A/L treatment: 5% or 7% prevalence of oocyst infection; both were effective relative to the control.

### Effect of chlorfenapyr on ***Plasmodium falciparum*** development

**Fig. 3:**
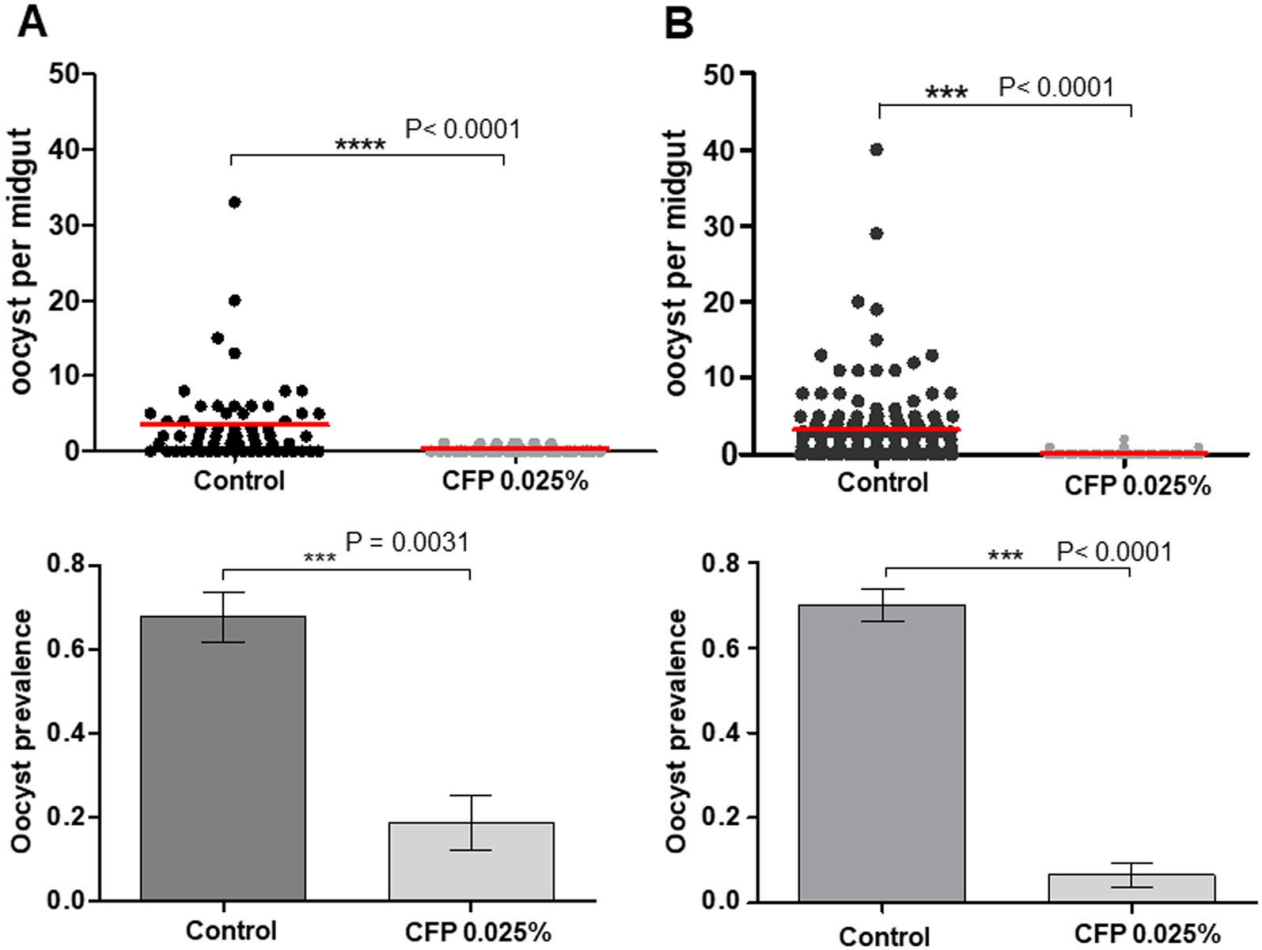
Prevalence of infection and oocyst intensity in *An. gambiae* s.s. exposed to ATSB solution (chlorfenapyr incorporated into ATSB) 15 h pre-infection (**A**) and 24 h post infection (**B**). CFP = Chlorfenapyr. Number of oocysts per female midgut is presented as a scatter dot plot for each treatment. Oocyst prevalence is presented as the bar chart, results are presented as mean ± standard error (s.e)

The second experiment investigated the impact of chlorfenapyr insecticide on parasites in mosquito midgut. Chlorfenapyr concentration (0.025%) was selected to induce < 25% of mosquito mortality (this is defined as the ‘sublethal dose’ in context of the experiment) in order to enable 80% mosquito survival and assess the treatment efficacy on *P. falciparum* development (Table 1). Figure 3A shows the prevalence of oocyst infection and intensity of infection in *An. gambiae* s.s. exposed to ATSB solution containing chlorfenapyr at 0.025% concentration prior to infection. Chlorfenapyr exposure significantly reduced oocyst prevalence and oocyst intensity both before and after infection (Fig. 3A). Infected mosquitoes exposed to chlorfenapyr had lower prevalence of infection (19% [9.2–34.9]) than unexposed mosquitoes (68% [55.0–78.3], OR [95% CI] = 0.1 [0.04–0.3], p < 0.0001) (Fig. 3A). A reduced oocyst load was observed in mosquitoes exposed to chlorfenapyr (mean oocyst in midgut = 0.18 ± 0.06) compared to the control (mean oocyst in midgut = 3.43 ± 0.6, p < 0.0001). When mosquitoes were exposed to a sublethal dose of chlorfenapyr (0.025% dose) after an infectious blood meal, a significant reduction of both infection rate and oocyst intensity were found: 90% reduction of oocysts prevalence (6.6% [2.7–14.9] in mosquito exposed to CFP vs. 67.1% [60.8–72.8] with control, OR [95% CI] = 0.03 [0.01–0.0.08], p < 0.0001) and oocyst intensity (mean = 0.07 ± 0.03 in mosquito exposed to CFP compared with 3.22 ± 0.42 in Control, U = 2094, P < 0.0001) (Fig. 3B).

**Table 1:** Proportion of *Anopheles gambiae* s.s. killed after 72 h in preliminary bioassays to determine a sublethal concentration of chlorfenapyr

Strain	CFP concentration %	Total tested	Total dead	% mortality after 72 h
	0.5	101	101	**100**
	0.25	93	77	**82.8**
M’BE G2	0.125	107	66	**61.7**
	0.05	91	41	**45.1**
	0.025	103	18	**17.5**
	0.0025	80	7	**8.7**
	0	106	3	**2.8**

### Effect of chlorfenapyr vs. anti-malarial on *Plasmodium falciparum* development

**Fig. 4:**
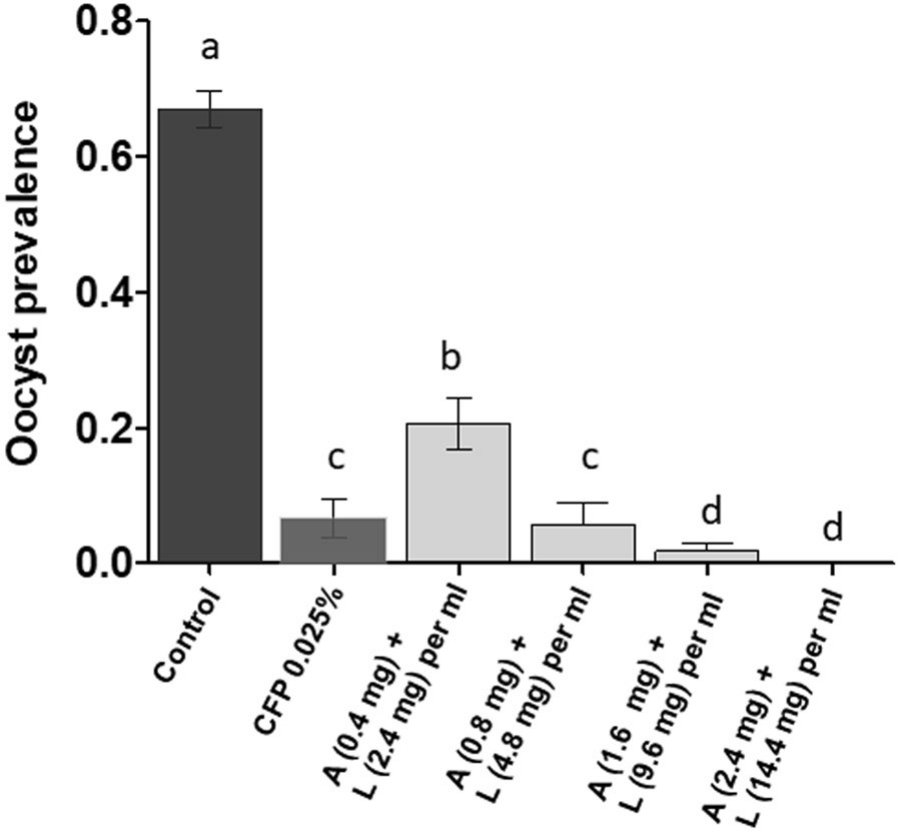
Prevalence of infection in *An. gambiae* s.s. exposed to ATSB solution (chlorfenapyr or differents doses of Artemether + Lumefantrine) 24 h post infection. CFP = Chlorfenapyr, A + L = Artemether + Lumefantrine. Results are presented as mean ± standard error (s.e). Different letters indicate significant differences

**Fig. 5:**
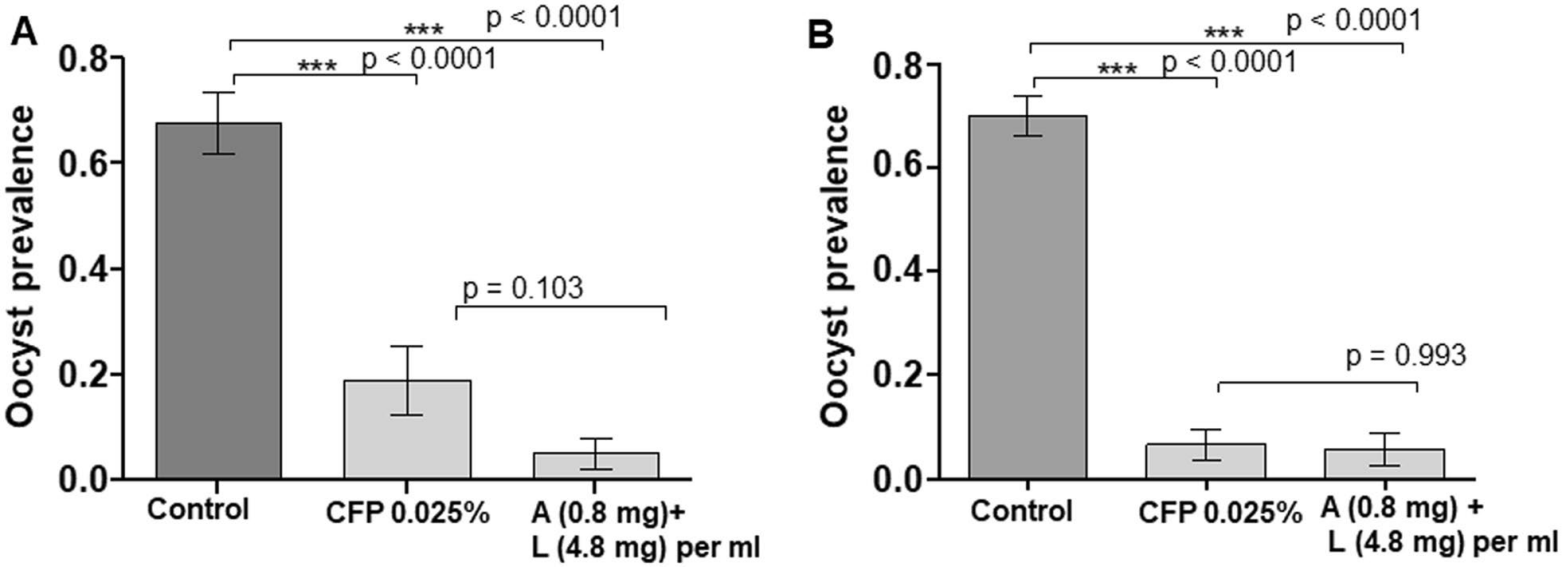
Prevalence of infection in *An. gambiae* s.s. exposed to ATSB solution 15 h pre-infection (**A**) and 24 h post infection (**B**). CFP = Chlorfenapyr, A + L = Artemether + Lumefantrine. Results are presented as mean ± standard error (s.e)

Sublethal chlorfenapyr dose induced significantly lower prevalence of infection than the lower anti-malarial dose (6.6% [2.7–14.9] for CFP vs. 24% [16.1–34.9] for A/L, OR [95% CI] = 0.22 [0.06–0.7], p < 0.012) (Fig. 4). The difference was no longer significant when the anti-malarial dose doubled either before or after infected blood meal (p > 0.05) (Fig. 5). Increasing A/L to 1.6/9.6 mg/ml and 2.4/14.4 mg per ml had detrimental effect on infection prevalence (84 and 100% reduction of infection prevalence, respectively) relative to the impact with chlorfenapyr (Fig. 4).

## Discussion

The midgut stages of parasite development constitute prime targets for strategies aiming to block malaria transmission. In this study, it was investigated whether anti-malarials or insecticides, incorporated into ATSB could affect the development of malaria parasite, *P. falciparum*, in wild *An. gambiae* s.s. pyrethroid resistant strains containing both metabolic and voltage-gated sodium channel (VGSC) *kdr* mechanisms of pyrethroid resistance. Female mosquitoes were fed on ATSB either before or after receiving infective blood meals to reveal vulnerable times of exposure to ATSB. The results showed that exposure to a range of concentrations of anti-malarial drugs significantly reduced the prevalence and intensity of *P. falciparum* oocysts in resistant *An. gambiae* s.s. Parasites were completely eliminated from the midguts of infected females exposed to A/L treatment (2.4/14.4 mg/ml), whereas females exposed to negative control ASB showed a high prevalence and intensity of infection. This is consistent with previous observations that show impact of anti-malarial drugs on *Plasmodium* development. It has, been demonstrated that the early midgut development of *P. falciparum* is arrested by exposing *An. gambiae* to surfaces treated with the anti-malarial drug atovaquone [23]. The development of parasites was also inhibited when mosquitoes were fed on mice infected with *Plasmodium berghei* and injected with the same anti-malarial drug [34]. Atovaquone is mitochondrial-acting drug that affects parasitic development in mosquitoes at the zygote–ookinete transition [23]. Artemether/lumefantrin drugs used in this study are known to have gametocytocidal effects [35] and their anti-malarial properties stem from interference with parasite transport proteins and disruption of parasite mitochondrial function [36]. The results indicated that these compounds also might act on early stage of *P. falciparum* in mosquito midgut. In this paired experiment, the anti-malarial A/L was compared, as positive control, to the pyrrole insecticide chlorfenapyr. Artemether/Lumefanthrin is not an insecticide. In this context, the two treatments are reducing the ‘vector competence’ of the mosquito, a term borrowed from insect immunology and the concept of increasing the mosquito’s refractoriness to infection. Depending of the dosages administered, the insecticide and the anti-malarial appear to interfere with vector competence of the mosquito to a comparable degree.

One of the most interesting findings is that exposure to chlorfenapyr insecticide reduced both prevalence and intensity of infection in *An. gambiae*. This aligns with recent studies showing that sublethal deltamethrin, DDT or bendiocarb inhibited development of *P. falciparum* in insecticide-resistant *An. gambiae* s.s. [21, 37]. Earlier experiments investigating *Plasmodium yoelii* and *P. falciparum* development in the Asian vector *Anopheles stephensi* also showed that sublethal exposure to pyrethroids inhibited development of oocysts in the midgut [38]. Strain differences and genetic factors, including insecticide resistance all affect competence to transmit *P. falciparum* [37] and *P. berghei* parasites [39]. Resistant strains seem less conducive to parasite development than susceptible strains [39].

In the past, the side-effects of vector-borne chemicals on malarial parasites was minor topic in pesticide research [21]. With the selection of high level pyrethroid resistance to insecticide treated nets, and the problem this presents for malaria control, research on the indirect effects of pyrethroid on *Plasmodium* was initially consigned to the sidelines. However, the last 2 years has led to revision in thinking. Pyrethroid resistance has led to the development of Dual-active ingredient (AI) LLIN. Some insecticides on nets have turned out to be good and some disappointing. The most promising net of all is the chlorfenapyr-pyrethroid net *Interceptor G2* which in two recent cluster randomised controlled trials has shown it to be a most effective Dual-AI LLIN for preventing malaria transmitted by pyrethroid resistant mosquitoes [40, 41]. Chlorfenapyr has many unique properties, foremost of which are its novel mode of action and its apparent lack of cross resistance to existing insecticide classes [31, 42–44]. Its efficacy against all known forms of insecticide resistance, and now with its *Plasmodium*-blocking properties revealed, perhaps this marks a start to understanding why chlorfenapyr-pyrethroid LLIN appear so much more effective than pyrethroid-only treated LLIN and PBO-pyrethroid treated LLIN against populations of insecticide resistant vectors [40, 41].

How parasites are being affected by chlorfenapyr inside the mosquito is less clear. Chlorfenapyr is a pro-insecticide which functions to uncouple oxidative phosphorylation in the mitochondria, resulting in disruption of ATP production and subsequent death of the insect [45, 46]. This insecticide may act directly on both the insect flight muscle and the parasite mitochondrial function via tarsal contact. It is also possible that other mechanisms such as oxidative stress induced by insecticide exposure may have interacted with prevention of *Plasmodium* development through higher reactive oxygen level and increased cytochrome P450 expression [47]. More studies are needed to fully understand the mechanisms of interactions between different insecticides and the parasite in the mosquito vector, and the role these have in modulating transmission by insecticide resistant vectors.

An important bridge made by the present paper is its contribution to explaining why chlorfenapyr-pyrethroid LLIN have proven effective in cluster randomized controlled trials in West and East Africa [40, 41]. It might be because chlorfenapyr kills mosquito and *Plasmodium* parasite equally well despite the modus operandi of chlorfenapyr being different by tarsal contact with LLINs and ingestion into insect midgut after feeding on ATSB. Chlorfenapyr kills *Plasmodium* parasites just as well as an anti-malarial, it kills mosquitoes better than any other insecticide known to malaria control because of the lack of cross resistance.

A limitation of the present study is the absence of insecticide-ATSB positive controls to compare their Plasmodium-killing effects, such as ATSB with pyrethroid insecticide or chlorfenapyr absorbed through the insect cuticle following tarsal contact with chlorfenapyr treated nets. Both positive controls would be worth exploring in their own right, in follow-up experiments, to confirm whether the effect of chlorfenapyr on *Plasmodium* is rare/unique among novel insecticides or is limited to the ATSB mode of delivery.

Together, these results reinforce the idea of targeting specifically infective mosquitoes, the agents responsible for malaria transmission. A promising method for this purpose is ATSB, a novel vector control approach that could interfere with parasite development inside the mosquito.

## Conclusion

The present study demonstrated that the use of ATSB could obstruct malaria transmission by interfering with parasite function or development inside the mosquito. The potential of the ATSB approach for delivery of new compounds for malaria control may make it an ideal preventive tool for controlling pyrethroid-resistant mosquitoes and preventing malaria parasite transmission. This study provided evidence for a new approach to reducing the global burden of malaria and potentially other mosquito vector-borne diseases. Whether or not ATSB will become a powerful tool for vector control in the future, the use of sugar baits in laboratory experiments has already led to greater understanding why chlorfenapyr mixed with pyrethroid in recent chlorfenapyr-pyrethroid LLIN trials nets is a potent combination.

## Data Availability

The datasets used and/or analysed during the current study are available at Institut Pierre Richet/Institut National de Santé Publique and will be made available on reasonable request.

## Abbreviations

ATSB: Attractive targeted sugar bait
A/L: Artemether/lumefantrine
CFP: Chlorfenapyr
LLINs: Long lasting insecticidal nets
IRS: Insecticide residual spraying
ITNs: Insecticide-treated nets
DDT: Dichloro-diphenyl-trichloroethane
ASB: Attractive sugar bait
